# The Quest to Model Chronic Traumatic Encephalopathy: A Multiple Model and Injury Paradigm Experience

**DOI:** 10.3389/fneur.2015.00222

**Published:** 2015-10-20

**Authors:** Ryan C. Turner, Brandon P. Lucke-Wold, Aric F. Logsdon, Matthew J. Robson, Matthew L. Dashnaw, Jason H. Huang, Kelly E. Smith, Jason D. Huber, Charles L. Rosen, Anthony L. Petraglia

**Affiliations:** ^1^Department of Neurosurgery, West Virginia University School of Medicine, Morgantown, WV, USA; ^2^Center for Neuroscience, West Virginia University School of Medicine, Morgantown, WV, USA; ^3^Department of Basic Pharmaceutical Sciences, West Virginia University School of Pharmacy, Morgantown, WV, USA; ^4^Department of Pharmacology, Vanderbilt University School of Medicine, Nashville, TN, USA; ^5^Department of Neurosurgery, University of Rochester School of Medicine and Dentistry, Rochester, NY, USA; ^6^Department of Neurosurgery, Baylor Scott and White Health System, Temple, TX, USA; ^7^Division of Neurosurgery, Rochester Regional Health, Rochester, NY, USA

**Keywords:** chronic traumatic encephalopathy, tauopathy, animal models, cognitive performance, perivascular pathology

## Abstract

Chronic neurodegeneration following a history of neurotrauma is frequently associated with neuropsychiatric and cognitive symptoms. In order to enhance understanding about the underlying pathophysiology linking neurotrauma to neurodegeneration, a multi-model preclinical approach must be established to account for the different injury paradigms and pathophysiologic mechanisms. We investigated the development of tau pathology and behavioral changes using a multi-model and multi-institutional approach, comparing the preclinical results to tauopathy patterns seen in post-mortem human samples from athletes diagnosed with chronic traumatic encephalopathy (CTE). We utilized a scaled and validated blast-induced traumatic brain injury model in rats and a modified pneumatic closed-head impact model in mice. Tau hyperphosphorylation was evaluated by western blot and immunohistochemistry. Elevated-plus maze and Morris water maze were employed to measure impulsive-like behavior and cognitive deficits respectively. Animals exposed to single blast (~50 PSI reflected peak overpressure) exhibited elevated AT8 immunoreactivity in the contralateral hippocampus at 1 month compared to controls (*q* = 3.96, *p* < 0.05). Animals exposed to repeat blast (six blasts over 2 weeks) had increased AT8 (*q* = 8.12, *p* < 0.001) and AT270 (*q* = 4.03, *p* < 0.05) in the contralateral hippocampus at 1 month post-injury compared to controls. In the modified controlled closed-head impact mouse model, no significant difference in AT8 was seen at 7 days, however a significant elevation was detected at 1 month following injury in the ipsilateral hippocampus compared to control (*q* = 4.34, *p* < 0.05). Elevated-plus maze data revealed that rats exposed to single blast (*q* = 3.53, *p* < 0.05) and repeat blast (*q* = 4.21, *p* < 0.05) spent more time in seconds exploring the open arms compared to controls. Morris water maze testing revealed a significant difference between groups in acquisition times on days 22–27. During the probe trial, single blast (*t* = 6.44, *p* < 0.05) and repeat blast (*t* = 8.00, *p* < 0.05) rats spent less time in seconds exploring where the platform had been located compared to controls. This study provides a multi-model example of replicating tau and behavioral changes in animals and provides a foundation for future investigation of CTE disease pathophysiology and therapeutic development.

## Introduction

Current limitations in understanding CTE pathophysiology are unlikely to be addressed in a clinical population in the near future due to the challenges associated with establishing long-term, prospective cohort studies in such a population. Preclinical rodent models serve to fill this gap in knowledge for various disorders and could allow for further investigation of the molecular mechanisms responsible for CTE, as well as testing the potential of diagnostic and therapeutic approaches under development. Few preclinical models of CTE have been proposed that sufficiently demonstrate both the requisite tauopathy and behavioral changes attributed to CTE (1–25).

The purpose of this paper is to present two preclinical models that successfully reproduce some neuropathological and behavioral changes consistent with CTE-like phenotypes, and discuss future directions for CTE animal modeling. Brody and colleagues (25) present several unanswered questions that we expand upon in our accompanying review such as the role of (1) inter-injury interval, (2) number of impacts, (3) impact severity, (4) age at time of impacts, (5) mechanism of impact, (6) genetics, (7) gender, and (8) effect of environment on the likelihood and/or progression of CTE development. We recently reported that endoplasmic reticulum stress might be a contributing factor linking acute neurotrauma to behavioral deficits (26). The quest for elucidating CTE pathophysiology development is ongoing with the goal of targeting specific injurious cascades a key priority in order to prevent the emergence of clinical symptoms.

In this work, we present novel models of neurotrauma-induced neurodegeneration in both mice and rats following exposure to single or repetitive brain injury, respectively. These models replicate both the tauopathy and some of the behavioral changes implicated in CTE in the clinical population. We believe the blast model is particularly relevant clinically due to utilization of a scaled, short-duration blast exposure. This is a striking contrast to long-duration blasts utilized in numerous studies that may more closely approximate an atomic blast than a blast from an improvised explosive device (27–30). Park and colleagues show that by having the animal outside of the tube, the wave is directed toward the skull causing neurologic injury without extensive lung injury (31). Extended duration waves are generated when the tube has disproportionate length and volume ratios for the driven and driver sections. When the animals are placed within a tube that has not been scaled, the impulse exposure to the skull might not be representative of human exposure (32). The modified closed-head injury in *unanesthetized* animals described previously by Petraglia and colleagues offers the benefit of injuring awake animals, more closely replicating the clinical picture seen in athletes than most other TBI models (23, 24). Further studies are required to address injury paradigms that do or do not contribute to CTE development but utilization of these models appears promising in not only modeling CTE but also identifying therapeutic targets based upon other recently published work by our groups (26, 33).

## Materials and Methods

### Animals and Human Samples

All experiments involving animals were approved by either the Institutional Animal Care and Use Committee of West Virginia University or that of the University of Rochester and were performed based upon principles of the *Guide for the Care and Use of Laboratory Animals*. Fifty six (56) young-adult male rats (300–350 g) were acquired from Hilltop Laboratories (Scottdale, PA, USA). All blast procedures were performed at West Virginia University. Twelve (12) young-adult male C57BL/6J mice were acquired from Jackson Laboratories (Bar Harbor, ME, USA) and used for modified controlled cortical impact at the University of Rochester. All animals were allowed to acclimate upon arrival for 1 week prior to any experimentation. At all times animals were provided food and water *ad libitum* and maintained on a 12-h light-dark cycle. Human samples were from deceased professional athletes that were previously diagnosed with chronic traumatic encephalopathy (CTE) (34). The tissue was collected from the entorhinal cortices.

### Experimental Groups

**Figure 1:**
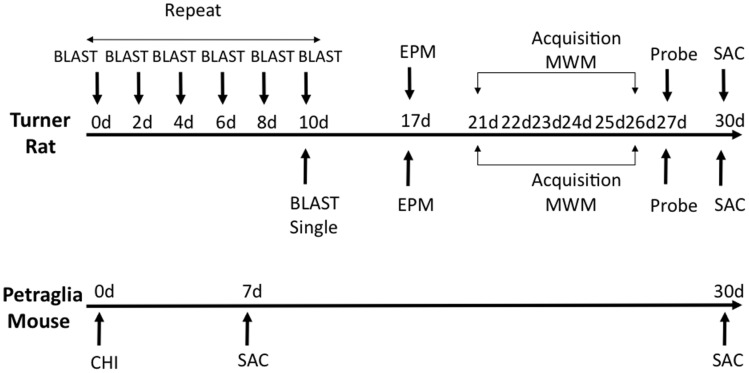
**Schematic showing experimental design and behavioral experiments**. Time of sacrifice for biochemical experiments is also shown.

Fifty six (*n* = 56) rats were divided into three primary experimental groups for behavior – anesthetized controls (*n* = 24), a single blast injury (*n* = 16), and repeat blast injuries (*n* = 16). Each of these groups was sacrificed at 1 month following the final blast or sham-injury and after undergoing functional assessment. Elevated-plus maze was done at 7 days post-blast (*n* = 24; eight controls, eight single blast, and eight repeat blast) and the Morris water maze started at 21 days post-blast (*n* = 32; 16 controls, 8 single blast, and 8 repeat blast). Following behavioral analysis, rats from the EPM group were divided into two separate groups with one utilized for immunohistochemistry (*n* = 10; four controls, three single blast, and three repeat blast) and the other for western blotting (*n* = 14; four controls, five single blast, and five repeat blast). Twelve (12) mice were divided into three primary experimental groups – anesthetized controls (*n* = 4), single injury with sacrifice at 1 week (*n* = 4), and single injury with sacrifice at 1 month (*n* = 4). All mice were utilized for western blotting techniques at time of sacrifice. An experimental schematic can be seen in Figure 1.

### Traumatic Brain Injury

**Figure 2:**
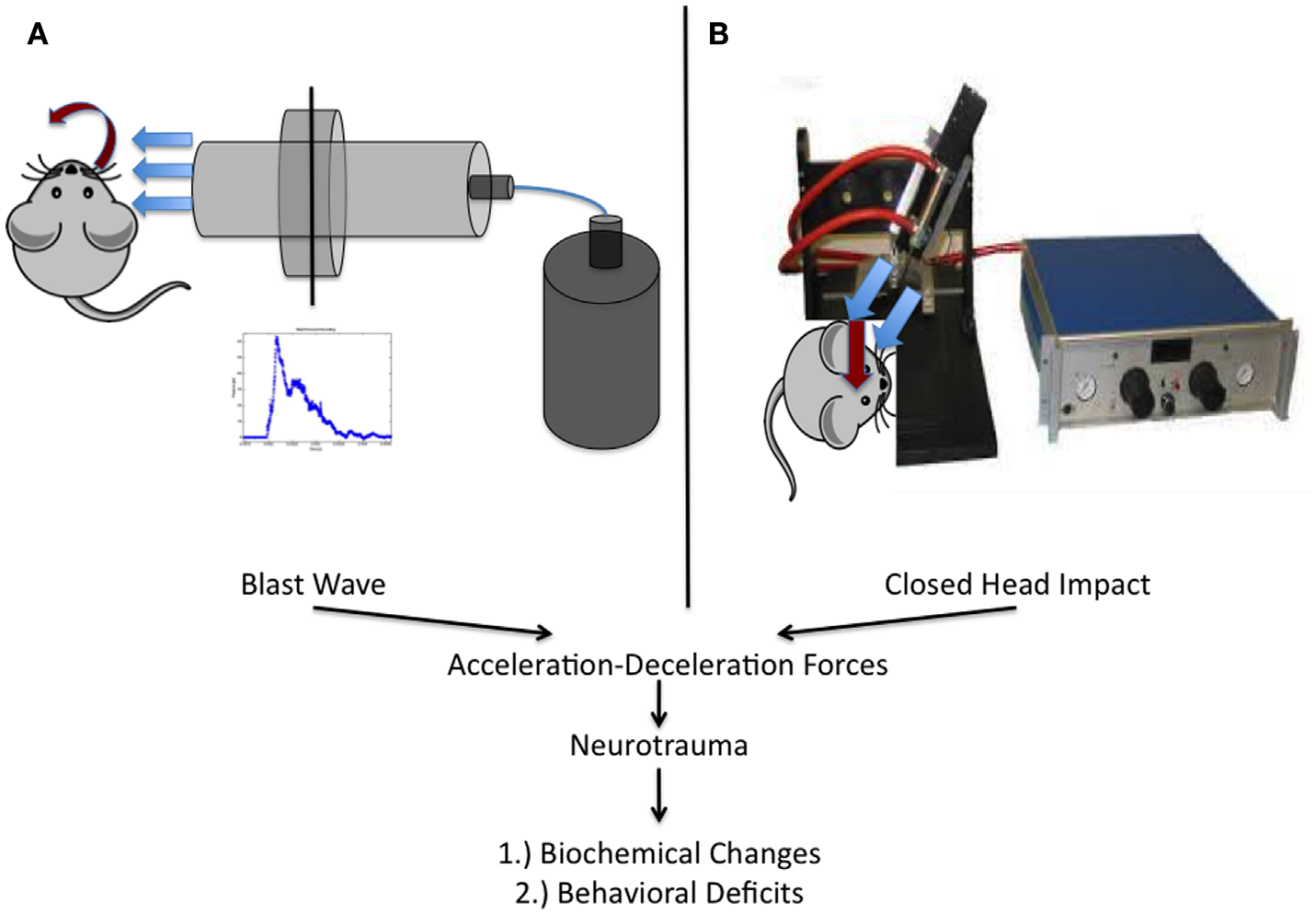
**Schematic representation of the two injury models utilized within this work**. **(A)** Experimental setup utilized at West Virginia University for the study of blast-induced neurotrauma. The shock tube consists of a high-pressure driver section (nitrogen gas filled) and a low-pressure driven section (ambient air filled). When the membrane dividing the chambers ruptures, the blast wave is formed and released, encountering the rat from the right side of the cranium. **(B)** Depiction of the model utilized at the University of Rochester in *unanesthetized* mice. An electromagnetic impactor used in CCI was modified with a rubber tip and a specially designed helmet was placed on the mouse as previously described.

Briefly, all blast injuries administered to rats as part of this work were of moderate intensity (~50 PSI peak reflected overpressure) as determined previously and completed under isoflurane-based anesthesia (26, 29, 33). Blast exposure occurred on the right side of the animal and was only administered to the head and neck region. The rat was outside of the blast tube and a rigid barrier protected the remainder of the animal. Blast waves were of a short-duration (~2 ms) to ensure clinical relevance based on elucidated scaling parameters (Figure 2A). The scaling parameters are based on impulse dynamic measurements, which are more representative of human blast than closed models based on duration (35).

Mice were utilized in a manner previously described (23, 24). Briefly, mice were placed, unanesthetized, into a rodent restraint bag/cone and immobilized on top of a foam bed of known spring constant. A helmet was secured to the head using an elastic band, allowing for administration of a diffuse impact through force distribution. The helmet is made of stainless steel and measures 3 mm in thickness and 6 mm in diameter. Impacts were delivered using a modified controlled cortical impact device that was adjusted to include an altered tip of vulcanized rubber (Figure 2B). The impact was zeroed so that it was directly perpendicular to the helmet surface and orthogonal to the skull. The tip was driven 1 cm past zero point with a 100 ms impact.

### Western Blot

Animals were anesthetized and sacrificed by rapid decapitation. Brains were extracted and immediately placed in a lysate buffer with protease and phosphatase inhibitors as described elsewhere (26). Brains were dissected and tissue flash frozen and stored at −80°C prior to blotting. Hippocampal protein samples were dissolved in 0.5 mL of 1% sodium dodecyl sulfate (SDS) prior to sonication and protein assay. Pre-cast 10% 12-well gels (Life Technologies, Carlsbad, CA, USA) were loaded with 30 μg of protein per well and run with 2× Lammeli buffer. Wet transfer was performed using nitrocellulose membranes (Bio-Rad, Contra Costa, CA, USA) at 60 V for 2.5 h. Primary antibodies against AT8 (Pierce; Rockford, IL, USA), AT270 (Pierce; Rockford, IL, USA), CP13 (kindly supplied by Dr. Peter Davies), and PHF-1 (kindly supplied by Dr. Peter Davies) were utilized and detected utilizing the corresponding secondary. Membranes were imaged using a LI-COR fluorescent scanner (LI-COR; Lincoln, NE, USA) and images converted to gray scale. Analysis was performed using background subtraction (Odyssey Processing Software, LI-COR) and values normalized to β-actin levels, resulting in a normalized intensity value.

### Immunohistochemistry

For immunohistochemistry preparation, animals were anesthetized using isoflurane and transcardially perfused with ice-cold physiologic saline followed by 10% formalin for a total of 10 min. Brains were extracted and placed in fresh 10% formalin for a minimum of 24 h prior to blocking and subsequent paraffin embedding. Sections were prepared in 6-μm thickness using a Leica RM2235 microtome (Leica Microsystems, Wetzlar, Germany). Staining was performed using standard protocols used within the field and previously by our laboratory, using the antibodies described above as well as thioflavin for detecting neurofibrillary tangles (NFTs) (10, 36, 37).

### Behavioral Assessments

Learning and memory was assessed using the Morris water maze. Spatial acquisition trials began at day 21 after the final blast exposure (in both single and repeat injury paradigms). The pool utilized was ~180 cm in diameter and filled with water at ambient temperatures (18–21°C). A platform (10 cm × 10 cm) was submerged 2.5 cm below the surface of the water. A series of objects was placed in the environment around the pool to provide visual cues for the animal during trials. The training paradigm (spatial acquisition) consisted of 6 days with a total of four trials occurring each day per animal. Animals were placed into the maze from four different locations each day (four trials) with a total of 2 min (maximum) allowed per trial. Upon finding the platform, animals were allowed 15 s for acquisition to occur. If unsuccessful in finding the platform, animals were placed on the platform at the conclusion of 2 min by the investigator. On the probe trial day (platform removed), animals were placed in the maze at a novel location and allowed to explore the maze for 1 min. Data were acquired using AnyMaze™ video tracking software (Stoelting Co., Wood Dale, IL, USA) throughout all studies which allows for acquisition of latency, distance, and speed data to be analyzed across maze regions/quadrants.

Impulsivity was determined, at 7 days after the final blast, as previously described using an elevated-plus maze and measuring exploratory behavior (26, 38, 39). The apparatus was placed at a height of 60 cm from the floor and consisted of two open and two closed arms, with open arms opposing one another and intersecting perpendicularly with the opposed closed arms. Each arm was 50 cm long by 10 cm wide. Open arms were surrounded by clear plastic edging ~1.5 cm high. Closed arms were encased with black walls 30 cm tall, creating a three-sided and comforting enclosure for the rodent. At the start of each 5-min trial, animals were placed in the middle of the intersecting arms facing an open arm prior to release. Animals were allowed to explore the apparatus for the duration of the trial. AnyMaze™ software was utilized to record the animals’ position, distance traveled, and entry pattern into various arms throughout the trial. An increased percentage of time spent in the open arms was considered a sign of impulsive behavior (26, 38, 39).

### Statistical Analysis

An observer blinded to experimental condition performed all data acquisition. One-way Analysis of Variance (ANOVA) was used for statistical analysis of all tests except spatial acquisition trials of the Morris water maze and immunohistochemical comparisons in which a two-way, repeated measures ANOVA and students *t*-test were utilized, respectively. Bonferroni *post hoc* comparison was used to determine differences between experimental groups on two-way ANOVA with repeated measures for spatial acquisition. For all other comparison’s, a Tukey’s *post hoc* was utilized. Analysis was completed using GraphPad Prism 5.0 (GraphPad Software, Inc., La Jolla, CA, USA). A *p* < 0.05 was considered statistically significant for all data analyzed.

## Results

### Neurotrauma Induces tau Hyperphosphorylation in Both Blast and Closed-Head Injury Models

To elucidate the effect of neurotrauma on the development of CTE-like neuropathology, animals were exposed to sham-injury, single-injury (either blast or modified closed-head), or repeat-injury (blast). Animals were first assessed for tau phosphorylation, using AT8 and AT270 antibodies, believed to be the initial precursor of NFT development. AT8 forms a single band in rats and a double band in mice (14). Conversely, AT270 forms a double band in rats, but only a single band in mice (40). The number of bands is indicative of calpain-dependent phosphorylation, which is regulated uniquely between rats and mice (41). Increased phosphorylation of tau was observed after blast exposure and modified closed-head injury but the location of the increase was model-dependent. Specifically, tau phosphorylation was observed in the contralateral hemisphere in rats exposed to repeat blast injury but only in the ipsilateral hemisphere, and only with the AT8 antibody, in mice receiving a single modified closed-head injury.

**Figure 3:**
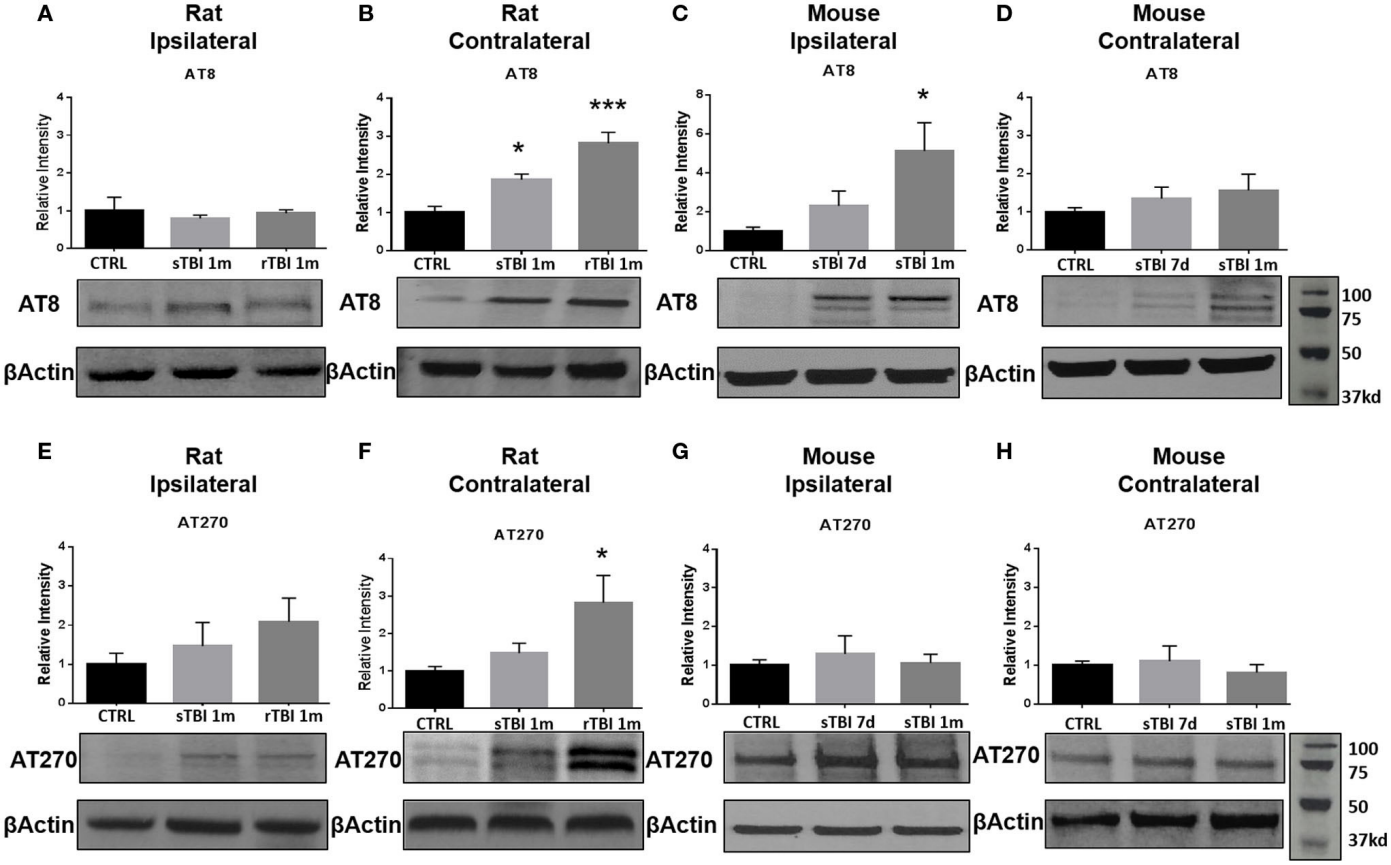
**Blast and closed-head injury models both induced tau hyperphosphorylation**. **(A)** Immunoblots show no significant difference in tau phosphorylation at serine sites 199/202 and threonine site 205 (AT8) at 1 month after single and repeat blast exposures in the ipsilateral rat hippocampus. **(B)** A significant increase in AT8 expression was measured after single (*p < 0.05 vs. CTRL) and repeat blast exposure (***p < 0.001 vs. CTRL) in the contralateral rat hippocampus. **(C)** AT8 expression was significantly increased at 1 month after closed-head injury in the ipsilateral mouse hippocampus (*p < 0.05 vs. CTRL). **(D)** No significant differences were observed in AT8 expression in the contralateral mouse hippocampus. **(E)** Immunoblots show no significant difference in tau phosphorylation at threonine site 181 (AT270) at 1 month after single and repeat blast exposure in the ipsilateral rat hippocampus. **(F)** A significant increase in AT270 expression was measured at 1 month after repeat blast exposure (*p < 0.05 vs. CTRL) in the contralateral rat hippocampus. **(G)** No significant differences were observed in AT270 expression in the ipsilateral mouse hippocampus, **(H)** or the contralateral mouse hippocampus after closed-head injury. One-way ANOVA Tukey’s *post hoc* analysis (values represent mean ± SEM; normalized to β-actin) (*n* = 3–5).

In rats exposed to blast, no significant difference in tau phosphorylation was observed in the ipsilateral hippocampus after blast injury for AT8 (Figure 3A) or AT270 (Figure 3E). A significant difference was observed in AT8 (*F*_2,11_ = 16.64, *p* < 0.001) and AT270 (*F*_2,9_ = 4.37, *p* < 0.05) levels in the contralateral hippocampus of rats exposed to blast injury. At 1 month following a single blast, a significant increase in tau phosphorylation recognized by AT8 increase was measured compared to control (*q* = 3.96, *p* < 0.05) (Figure 3B). A significant increase in phosphorylation detected by AT8 was also observed after repeat blast (*q* = 8.12, *p* < 0.001) (Figure 3B).

In the modified closed-head injury model utilized on the mice, a significant difference was observed in tau phosphorylation AT8 (*F*_2,9_ = 4.93, *p* < 0.05) in the ipsilateral hippocampus. At 1-month following injury, AT8 expression was elevated in comparison to sham-injury (*q* = 4.34, *p* < 0.05) (Figure 3C). No significant difference was observed at 7-days post-injury on the ipsilateral side or between any groups or time points on the contralateral side (Figure 3D).

At 1-month following repetitive blast exposure, a significant increase in phosphorylation recognized by the AT270 antibody was measured in the contralateral hippocampus of rats compared to anesthetized control animals (*q* = 4.03, *p* < 0.05) (Figure 3F). No significant differences were observed in tau phosphorylation recognized by AT270 in either ipsilateral (Figure 3G) or contralateral (Figure 3H) hippocampus of mice receiving modified closed-head injury.

### Hyperphosphorylation of tau Following Neurotrauma Occurs in Perivascular Brain Regions

**Figure 4:**
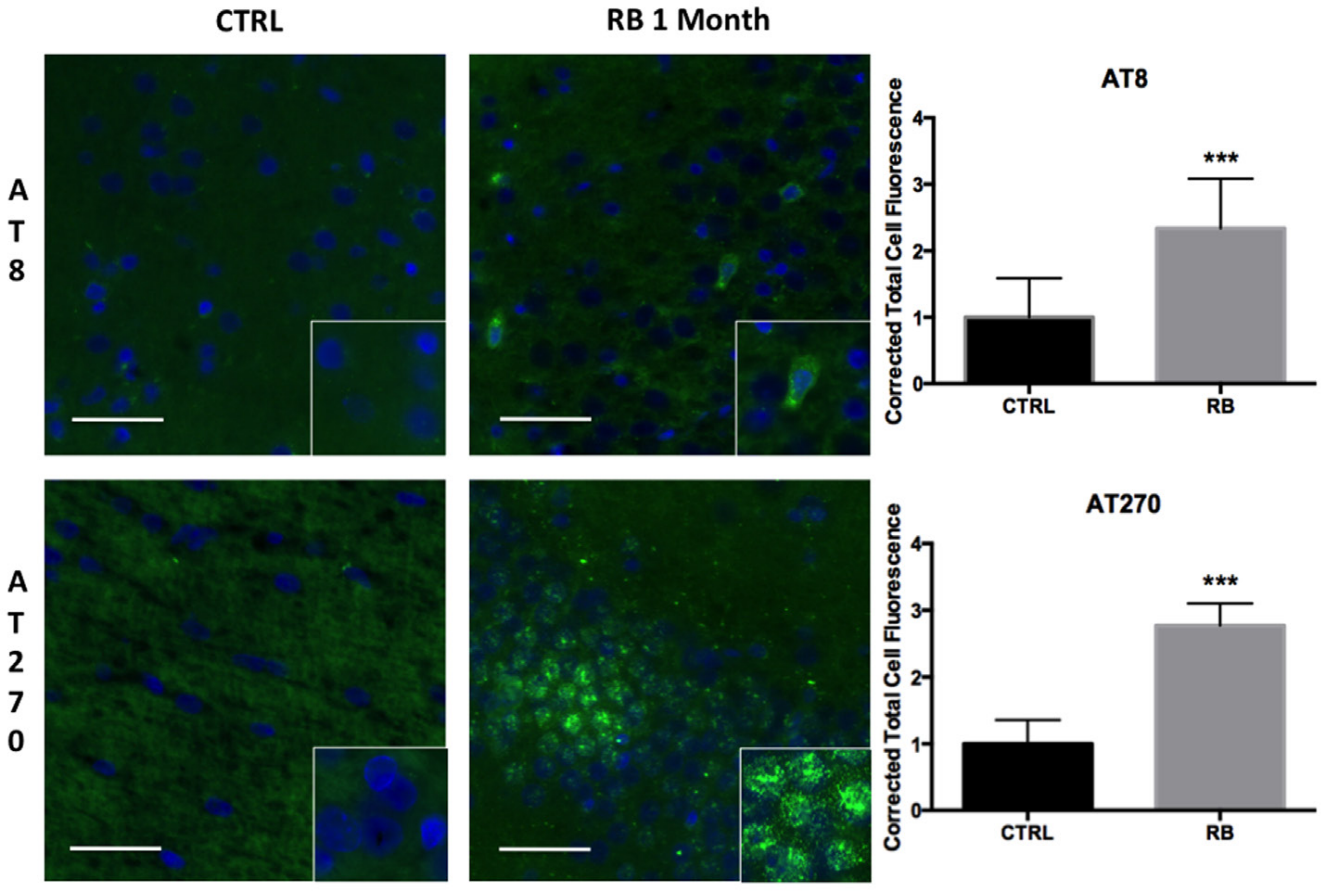
**Tau hyperphosphorylation is seen in microfoci throughout the contralateral superficial cortex**. AT8 was significantly increased at 1 month post repetitive blast (*t* = 4.455, *p* < 0.001). AT270 was also significantly increased following repetitive blast (*t* = 11.47, *p* < 0.001). AT270 was increased in a circular distribution. Tau hyperphosphorylation is an indicator of progressive pathology. Values were calculated using a student’s *t*-test comparing the mean difference between groups.

Clinical case series documenting patients with CTE have demonstrated the deposition of NFTs, an end product of tau hyperphosphorylation, in perivascular regions, which is a distinct difference from other tauopathies such as Alzheimer’s disease (AD). Possible mechanisms leading to NFTs in perivascular regions include the disruption of the blood–brain barrier (BBB), leading to punctate microhemorrhages, red blood cell breakdown, oxidative stress, and finally, persistent neuroinflammation. We observed a significant difference in tau hyperphosphorylation within perivascular regions of the contralateral hippocampus in rats exposed to repeat blast in comparison to control when identified with both AT8 (*t* = 4.46, *p* < 0.001) and AT270 (*t* = 11.47, *p* < 0.001) at 1-month post-injury (Figure 4). These findings were consistent with prior reports of CTE in humans and from our collection of post-mortem human samples with documented CTE.

### Neurotrauma is Associated with Conformational Changes in tau that are Recognized Precursors of Neurofibrillary Tangle Formation

**Figure 5:**
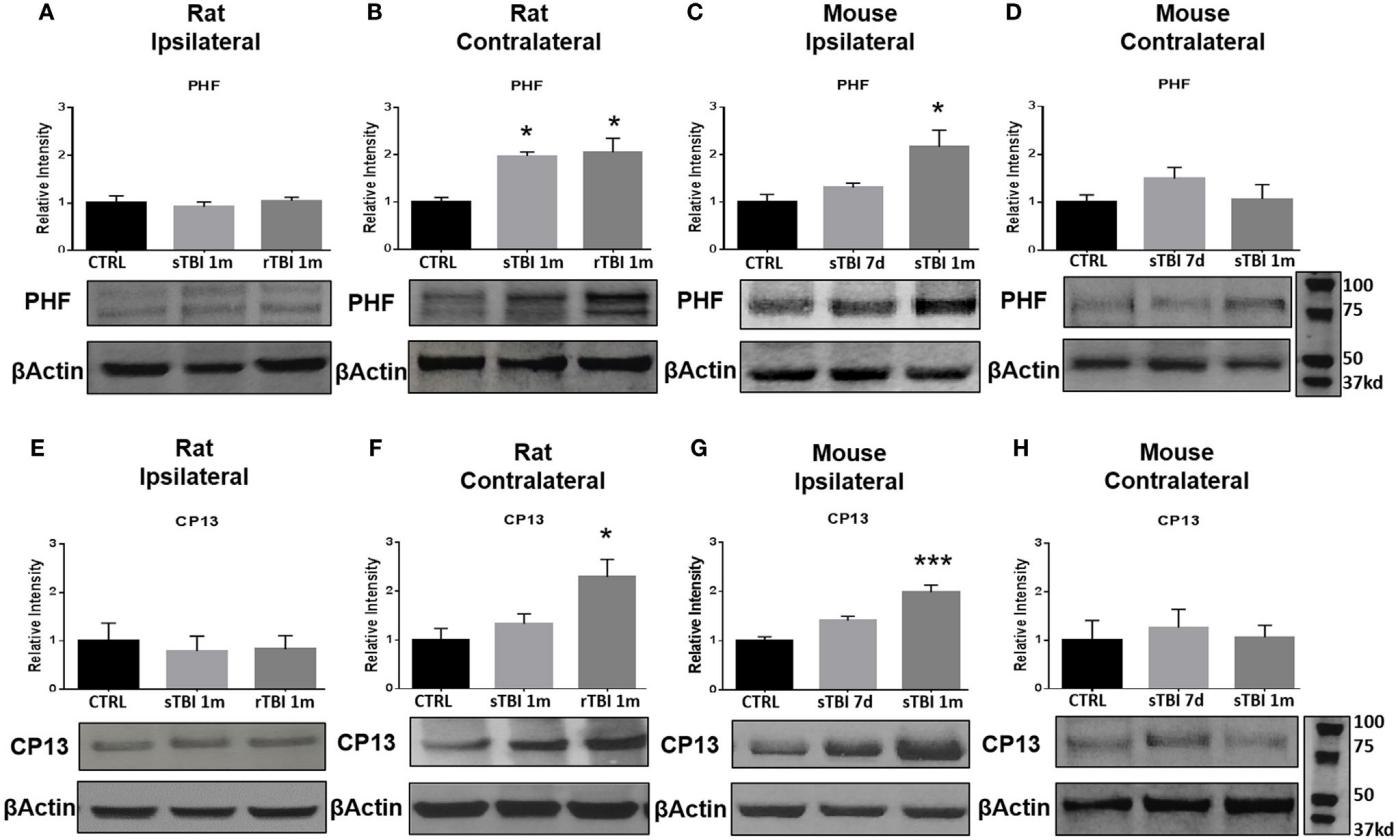
**Blast and closed-head injury models both induce conformational changes in tau proteins**. **(A)** Immunoblots show no significant difference in markers of tau conformational change at serine site 396/404 (PHF) at 1 month after single and repeat blast exposure in the ipsilateral rat hippocampus. **(B)** A significant increase in PHF expression was measured after single (*p < 0.05 vs. CTRL) and repeat blast exposure (*p < 0.05 vs. CTRL) in the contralateral rat hippocampus. **(C)** PHF expression was significantly increased at 1 month after closed-head injury in the ipsilateral mouse hippocampus. **(D)** No significant differences were observed in PHF expression in the contralateral mouse hippocampus. **(E)** Immunoblots show no significant difference in markers of tau conformational change at serine site 202 (CP13) at 1 month after single and repeat blast exposure in the ipsilateral rat hippocampus. **(F)** A significant increase in CP13 expression was measured at 1 month after repeat blast exposure (*p < 0.05 vs. CTRL) in the contralateral rat hippocampus. **(G)** A significant increase in CP13 expression was measured at 1 month after closed-head injury in the ipsilateral mouse hippocampus (***p < 0.001 vs. CTRL). **(H)** No significant differences were observed in CP13 expression in the contralateral mouse hippocampus after closed-head injury. One-way ANOVA Tukey’s *post hoc* analysis (values represent mean ± SEM; normalized to β-actin) (*n* = 3–5).

Following hyperphosphorylation, tau is purported to undergo conformational changes associated with subsequent insolubility and deposition/precipitation in the form of NFTs. Following neurotrauma in rats and mice, we found an elevation in markers of conformational change of the tau molecule based on immunoblotting with PHF-1 and CP13. After blast exposure, markers of tau conformational change were observed in the contralateral hemisphere of rats, while after the impact procedure in mice, the markers were found ipsilaterally. No significant differences in markers of tau conformation change were observed in the ipsilateral hippocampus after blast injury for neurofibrillary precursor PHF-1 (Figure 5A) or CP13 (Figure 5E).

A significant difference was observed in PHF-1 [*F*_(2,11)_ = 7.92, *p* < 0.01] and CP13 [*F*_(2,9)_ = 6.03, *p* < 0.05] levels in the contralateral hippocampus of rats exposed to blast injury. At 1 month following a single blast, a significant increase in PHF expression was measured compared to control (*q* = 4.76, *p* < 0.05) (Figure 5B).

A significant difference was observed in PHF-1 [*F*_(2,9)_ = 7.18, *p* < 0.05] levels in the ipsilateral hippocampus of mice exposed to injury. At 1 month following modified impact, a significant increase in PHF-1 expression was measured compared to control (*q* = 5.17, *p* < 0.05) (Figure 5C). No significant differences were observed in PHF-1 expression in the contralateral hippocampus of mice exposed to cortical impact (Figure 5D).

At 1 month after repetitive blast exposure, a significant increase in CP13 expression was measured in the contralateral hippocampus of rats compared to control (*q* = 4.73, *p* < 0.05) (Figure 5F). A significant difference was observed in CP13 [*F*_(2,9)_ = 21.70, *p* < 0.001] levels in the ipsilateral hippocampus of mice exposed to injury. At 1 month following impact, a significant increase in CP13 expression was measured compared to control (*q* = 9.28, *p* < 0.001) (Figure 5G). No significant differences were observed in CP13 expression in the contralateral hippocampus of mice exposed to cortical impact (Figure 5H).

**Figure 6:**
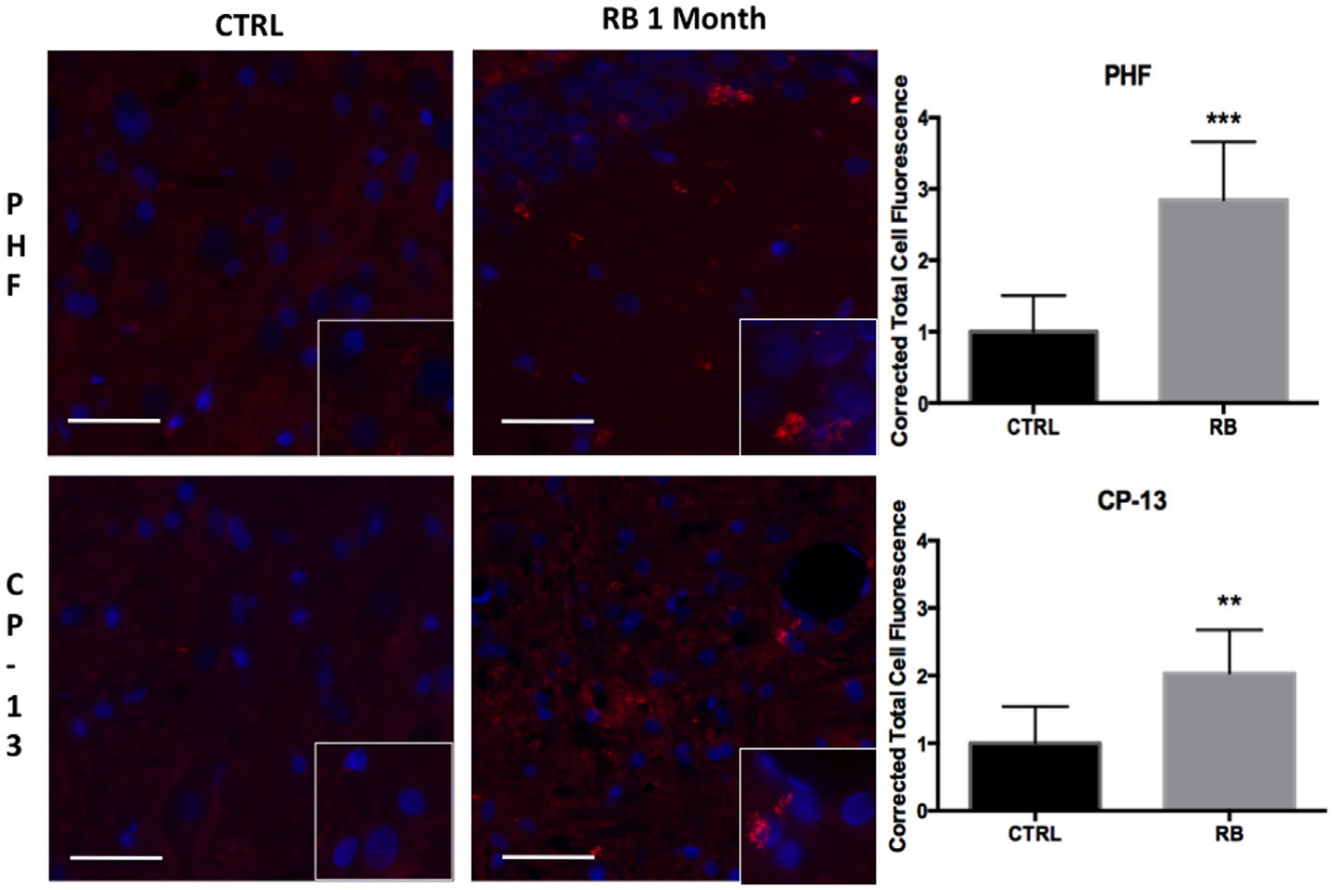
**Tau conformational markers were increased throughout the contralateral superficial cortex**. PHF was significantly increased at 1-month post repetitive blast (*t* = 6.055, *p* < 0.001). CP-13 staining was also significantly increased following repetitive blast (*t* = 3.883, *p* < 0.01). CP-13 was increased in a perivascular distribution. Tau conformational change is required for the formation of neurofibrillary tangles. Values were calculated using a student’s *t*-test comparing the mean difference between groups.

**Figure 7:**
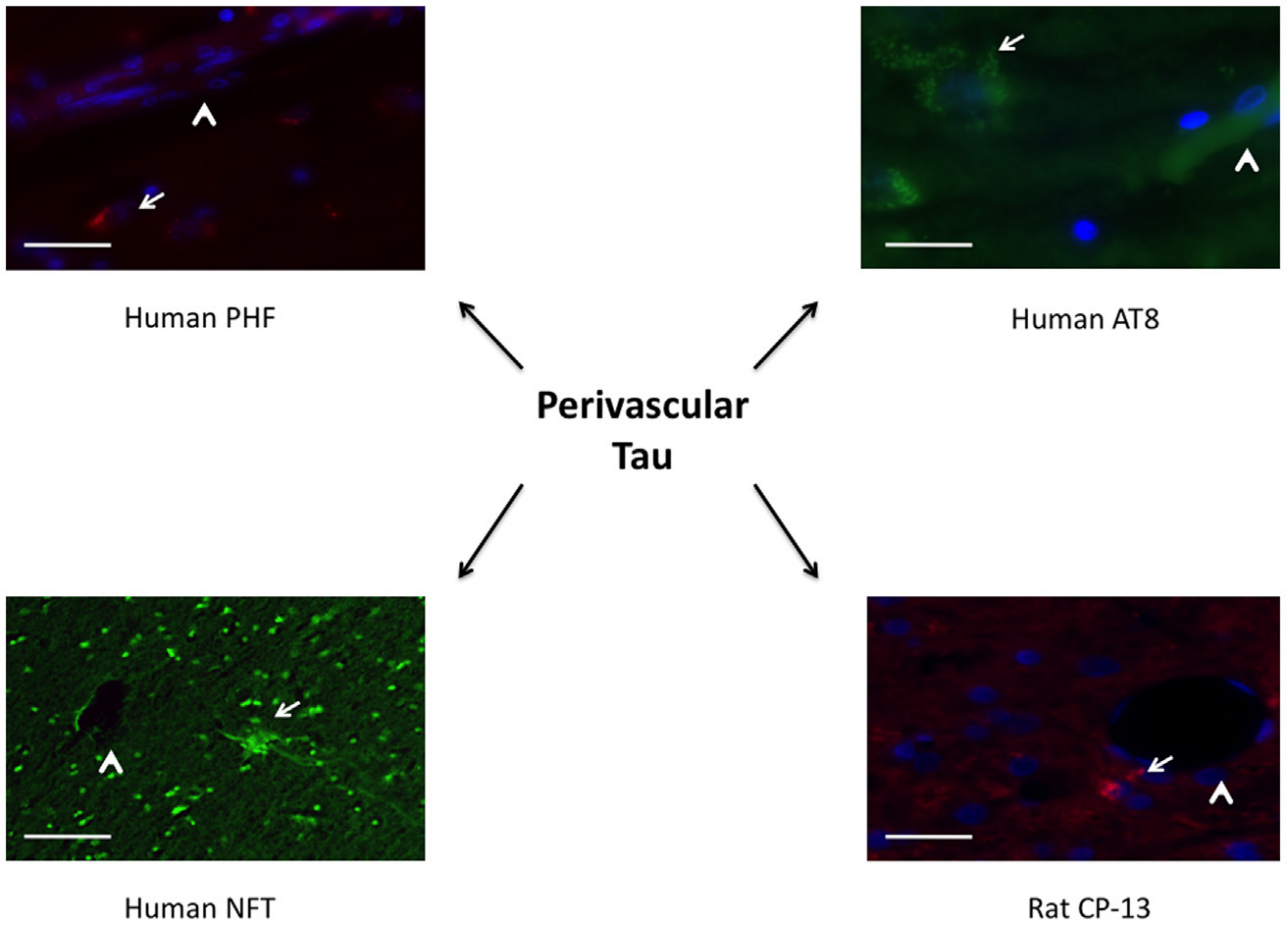
**Tauopathy is seen in a perivascular distribution**. PHF and AT8 were increased adjacent to longitudinal microvessels in human patients diagnosed with chronic traumatic encephalopathy. A neurofibrillary tangle stained with thioflavin was seen adjacent to a cut vessel lumen in the brain of a retired professional football player. Similarly, CP-13 was increased next to a cut vessel lumen in the contralateral cortex 1 month following repeat blast in the rat. Arrows indicate tauopathy while arrow heads point out vessels.

Similar findings were seen using immunohistochemistry when comparing repeat-injured animals to sham-injured animals at 1 month post-injury. Specifically, a significant difference was seen on PHF-1 staining (*t* = 6.06, *p* < 0.001) and CP-13 staining (*t* = 3.88, *p* < 0.01) (Figure 6). Again, the distribution of the staining was notable for being perivascular in nature, a finding shared across the clinical specimens diagnosed with CTE as seen in Figure 7. We show that PHF, AT8, and a thioflavin-stained NFT are increased perivascularly in human CTE specimens. CP-13 is increased in a perivascular distribution following repeat blast in a rat.

### Neurotrauma Produces Cognitive Impairments

**Figure 8:**
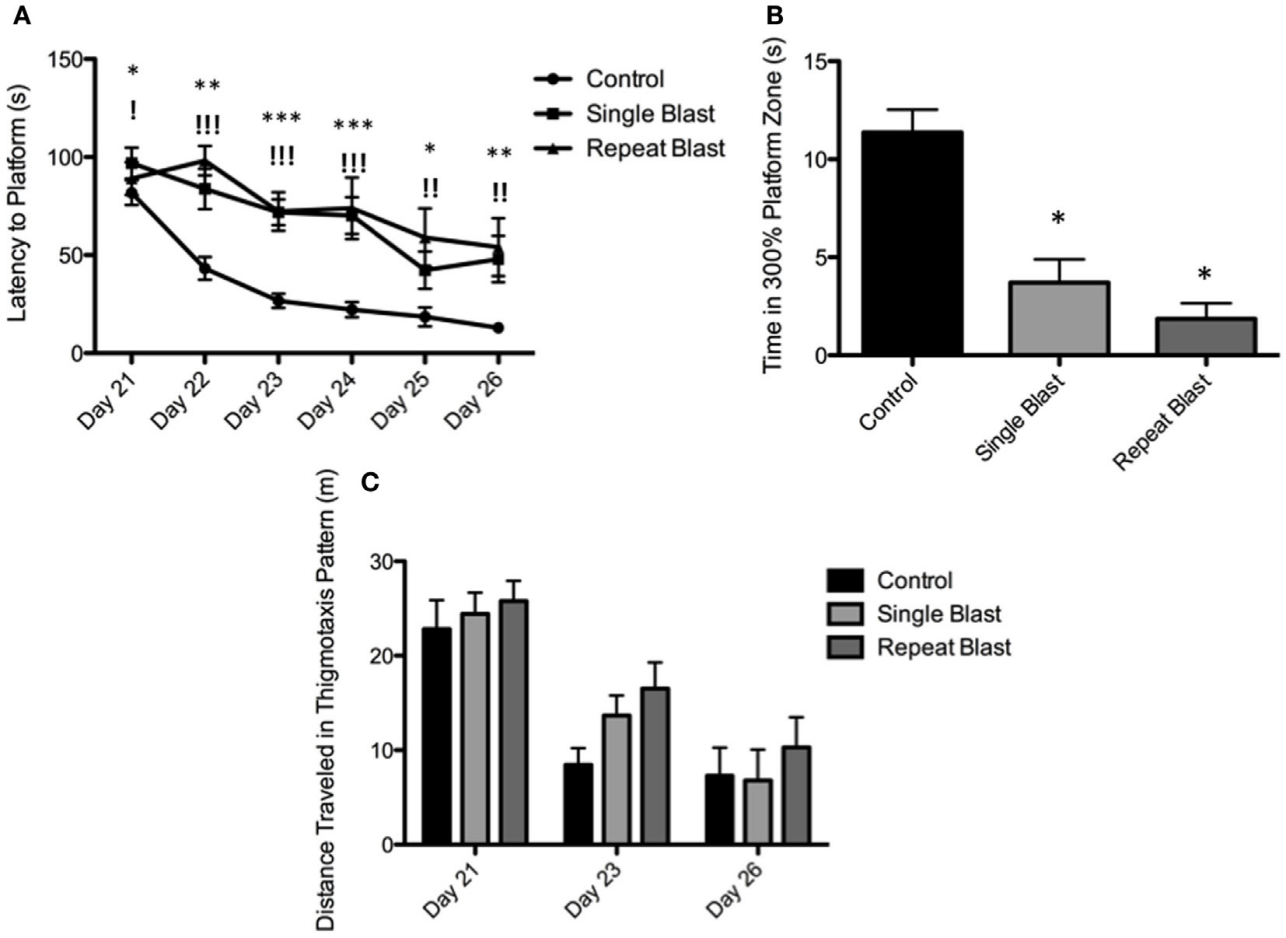
**Blast-induced brain injury produces deficits in learning and memory in rats**. **(A)** Spatial acquisition trials conducted on days 21–26 post-injury revealed deficits in learning in single-injured animals on days 22–24 and 26 in comparison to control. Repeat-injury was associated with deficits on days 22–26 in comparison to control. No differences were seen between single- and repeat-injury paradigms. **(B)** The probe test on day 27 revealed a statistically significant difference in time spent near the prior platform location between injured animals (single or repeat) and control animals. No difference was seen between single- and repetitively injured rats. **(C)** Animals exposed to neurotrauma exhibited a trend toward increased thigmotaxis during spatial acquisition procedures but this finding did not reach statistical significance.

**Figure 9:**
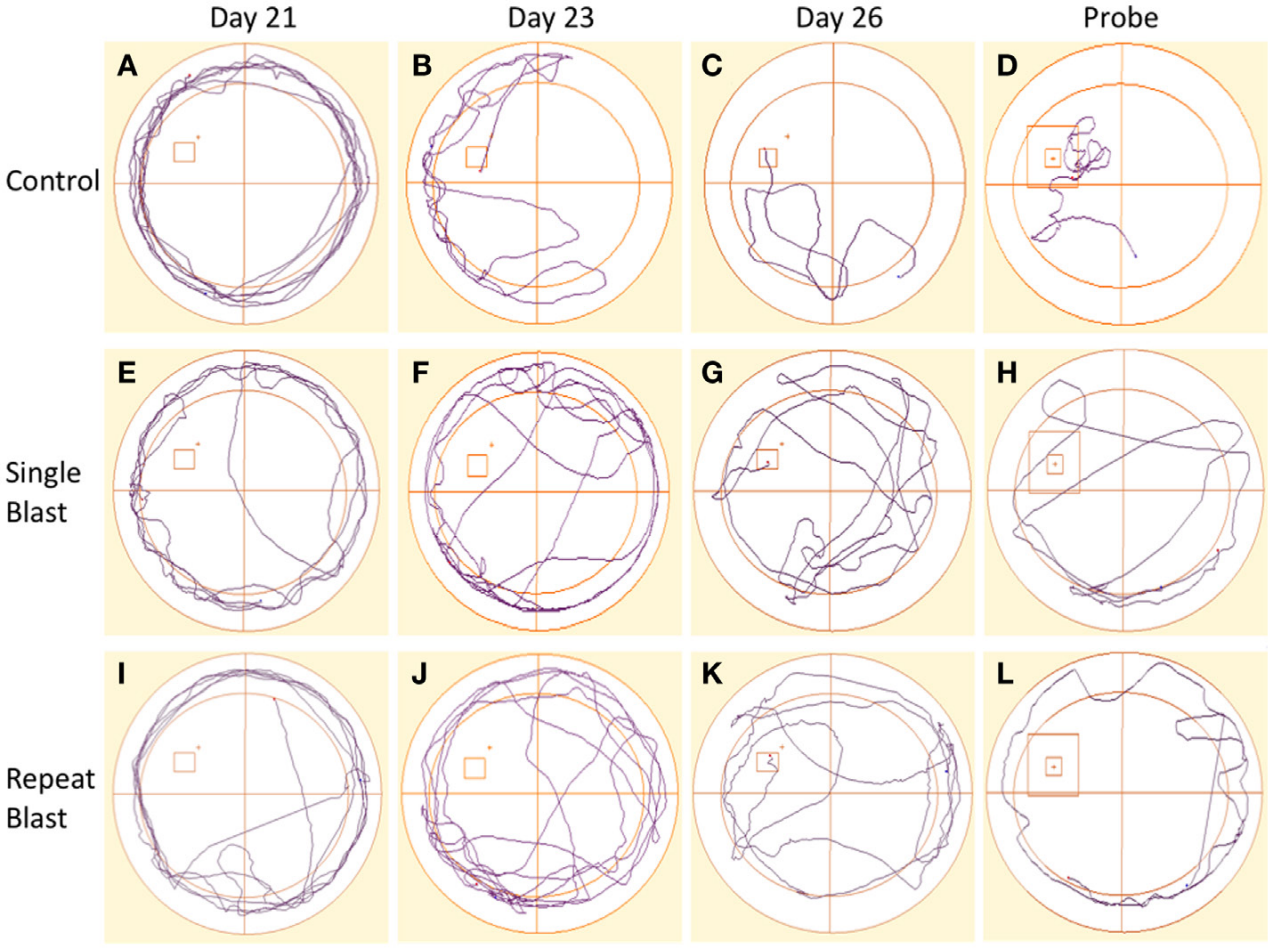
**Track plots acquired across spatial acquisition and probe testing in rats**. **(A–D)** Track plots generated from anesthetized control animals on days 21, 23, 26, and during the probe test. **(E–H)** Track plots generated from single-injured animals on days 21, 23, 26, and during the probe test post-injury. **(I–L)** Track plots generated from repetitively injured animals on days 21, 23, 26, and during the probe test post-injury. Injured animals appear to spend more time exhibiting thigmotaxis behaviors (circling edge of pool) on later days and in the probe trial fail to spend as much time as the anesthetized control animals within the probe region and the direct area surrounding the probe region.

Cognitive deficits have long been associated with the neuropathological diagnosis of CTE in the clinical population, particularly amongst the population diagnosed with CTE at a later age in life. Similarly, cognitive impairments in both learning and memory have been associated with neurotrauma but have not been presented in the context of a corresponding tauopathy in both a single and repeat blast injury paradigm. In cohorts of animals subjected to either sham-, single-, or repetitive-injury, spatial acquisition (learning) was assessed 3 weeks after the injury in single-injury animals and the final injury in repeat-injury paradigms (days 21–27 post-injury). Blast injury exposure was associated with worsened performance in the Morris water maze as evident by latency to find the platform in seconds when analyzed using a two-way repeated measures ANOVA. *Post hoc* tests revealed significant differences between anesthetized controls and single-injured animals during spatial acquisition on days 22–24, and day 26 (day 21: *t* = 1.38, *p* > 0.05; day 22: *t* = 3.70, *p* < 0.01; day 23: *t* = 4.12, *p* < 0.001; day 24: *t* = 4.38, *p* < 0.001; day 25: *t* = 2.17, *p* > 0.05; day 26: *t* = 3.20, *p* < 0.01). Similarly, *post hoc* tests demonstrated a significant impairment in acquisition between anesthetized controls and repetitively injured animals on days 22–26 (day 21: *t* = 0.66, *p* > 0.05; day 22: *t* = 5.01, *p* < 0.001; day 23: *t* = 4.16, *p* < 0.001; day 24: *t* = 4.72, *p* < 0.001; day 25: *t* = 3.69, *p* < 0.01; day 26: *t* = 3.76, *p* < 0.01). Notably, no differences were seen between single- and repeat-injured animals during the course of acquisition trials, despite the notable difference in tau pathology at this time presented earlier (Figure 8). The differences in latency to platform between blast-injured animals and anesthetized control animals is apparent as well based on visualization of the track plots recorded during data acquisition (Figure 9). Clearly different swimming patterns emerge with blast-injured animals exhibiting what appears to be more thigmotaxis (circling around the outer edge of the pool) than anesthetized control animals that reached statistical significance between controls and repeat-injured animals on day 23 (*q* = 3.58, *p* < 0.05), although this data point did not reach significance on days 21 and 26 based on automated measurements generated using AnyMaze™ (Figure 8).

Memory, as measured during the probe test (time spent in area surrounding the now-removed platform) conducted on day 27, again demonstrated deficits in animals subjected to blast-induced neurotrauma when analyzed using a one-way ANOVA (*F*_2,29_ = 20.01, *p* < 0.0001). *Post hoc* tests showed significant differences between sham and single injury animals (*t* = 6.44, *p* < 0.05) as well as between sham and repetitively injured animals (*t* = 8.00, *p* < 0.05) but no difference between single injury and repeat injury paradigms (Figure 8). These findings were confirmed visually using track plots generated during data acquisition (Figure 9). Anesthetized control animals exhibit a greater preponderance of pool crossings and swimming behavior within the region near the platform in contrast to blast-injured animals (Figure 9).

### Impulsivity is Increased Following Neurotrauma

**Figure 10:**
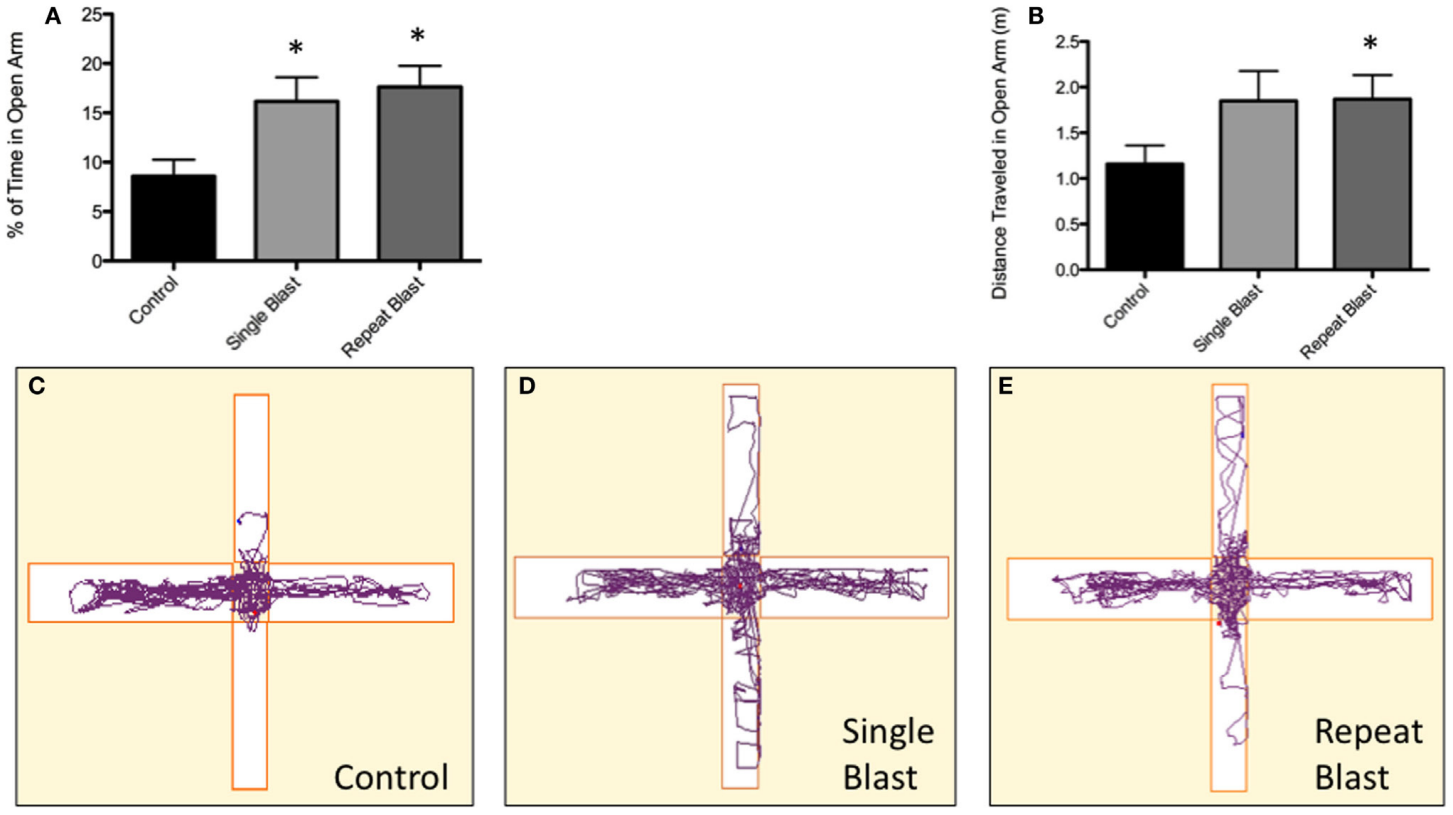
**Blast-induced brain injury in rats produces an increase in impulsive behavior based on the elevated-plus maze**. **(A)** A significant difference (*p* < 0.05 vs. CTRL) was seen between anesthetized control animals and both single and repeat blasts with regards to the percentage of time spent in the open arm of the elevated-plus maze. No difference was measured between single and repeat-injury paradigms. **(B)** While not reaching statistical significance, a trend toward greater distance traveled in open arms was seen with neurotrauma, a finding consistent with the documented increase in time spent in the open arms. **(C)** Animals subjected to neurotrauma appeared to travel less distance in the closed arms of the maze, although this finding did not reach statistical significance. **(D)** Track plot showing little exploration of the open arm by control animals. **(E)** Track plot showing increased exploration of the open arms of the elevated-plus maze by single-injured animals. **(F)** Track plot showing increased exploration of the open arms of the elevated-plus maze by repetitively injured animals.

Clinically, impulsive behavior has been described extensively in those with a history of repetitive neurotrauma and the context of CTE. To make an analogous comparison between our animal model of blast-induced traumatic brain injury and clinically reported symptoms, the elevated-plus maze was utilized to measure impulsive-like behaviors based upon the percentage of time spent in the open arms of the maze. Significant differences between groups were observed when analyzed using a one-way ANOVA (*F*_2,32_ = 5.03, *p* < 0.05). *Post hoc* tests revealed a significant difference between sham and single injury animals (*q* = 3.53, *p* < 0.05) as well as between sham and repetitively injured animals (*q* = 4.21, *p* < 0.05). No difference was seen between single and repeat injury paradigms with regards to percentage of time spent in the open arms of the apparatus (Figure 10). While not statistically significant, a trend toward a greater distance traveled in the open arms was observed with increasing levels of neurotrauma, evidence consistent with the percentage of time spent in the open arms (Figure 10). Conversely, a trend was also present regarding distance traveled in the closed arms with animals subjected to neurotrauma traveling less distance (Figure 10). *Post hoc* tests revealed no statistical significance when comparing sham to single injury groups but significance was reached when comparing sham to repetitively injured animals (*F*_2,33_ = 3.06; *q* = 3.47, *p* < 0.05).

## Discussion

While the clinical literature has been inundated with reports of CTE in athletes and soldiers alike, few experimental tools exist for investigating disease pathophysiology, establishing diagnostic criteria, and discovering preventative or therapeutic agents. For these reasons, preclinical models of CTE are highly desirable. In this work, we demonstrate two preclinical models capable of generating some biochemical and behavioral hallmarks of CTE, namely tauopathy and impulsive-like behavior. Furthermore, this work illustrates the utility of two distinct models and injury paradigms, namely blast exposure vs. a more traditional modified closed-head injury, and repeat vs. single injury paradigms respectively. Likewise, the fact that this work was completed in two different species and builds upon prior work by both West Virginia University and the University of Rochester laboratories validates the use of both mice and rats in the preclinical modeling of neurotrauma-related neurodegeneration (23, 24, 26). The modified closed-head injury represents a helmeted design with controlled placement of the impact likely accounting for ipsilateral deficit. The tauopathy development period following modified controlled impact was not previously elucidated therefore warranting the 7-day time point. Petraglia and colleagues show that astrocyte activation and cell death occurs at 7 days post modified controlled impact (24). We previously reported that tauopathy following blast in rats does not develop until weeks after injury and is on the contralateral side due to coup/contra-coup injuries (42).

The potential utility of these, as well as other preclinical models of neurotrauma-related neurodegeneration, is highly promising for investigation of disease pathophysiology, particularly as related to biochemical endpoints associated with CTE. To fully develop a CTE model, transgenic rodents will be needed that include amyloid, tau, and TDP43 pathology. The quest for elucidating the underlying mechanisms behind CTE development is ongoing. In previous work, we have shown blast causes substantial blood brain barrier disruption (33), endoplasmic reticulum stress activation (26), and oxidative stress (43). We show in this work the induction of hyperphosphorylated tau, conformational changes in tau, and more advanced precursors of NFT formation with the usage of CP13 and PHF-1 antibodies. AT8 binds to serine 199 and 202 as well as threonine 205. AT270 binds to threonine 181, PHF binds to serine 396 and 404, and CP13 binds to serine 202. Serine 396 and 202 are only exposed after tau undergoes conformational change (20). These changes are the ultimate result of tau hyperphosphorylation and protein misfolding/aggregation, and are likely related to dysregulation of the tau kinase/phosphatase system (20). Peclinical modeling of neurotrauma-related neurodegeneration will allow for the elucidation of how and when these kinases and phosphatases become dysregulated, and will allow for increased understanding of the disease process. Additionally, these studies will provide targets for direct or indirect therapeutic development. Similarly, preclinical models may prove instrumental in identification of diagnostic and prognostic tests for establishing a diagnosis of CTE and tracking disease progression. There is a clinical need for a rapid, cheap, and reliable diagnostic test to predict severity of CTE. Currently neuropathological examination remains the gold standard for diagnosis with some studies purporting the use of PET-based imaging for diagnosis (44–46). It is possible that development and validation of these techniques, and others such as diffusion tensor imaging and magnetic resonance spectroscopy may be accelerated through the application to preclinical models. The current limitation is that advanced imaging is expensive, cumbersome, and requires expertise from subspecialty radiologists.

Despite this work illustrating what we believe represents a clear step forward in the study of neurotrauma-related neurodegeneration, it is clear that further study is warranted. Preclinical models must be utilized to more fully characterize behavioral, biochemical, imaging, and electrophysiological/functional changes associated with the development of CTE. Future studies will likely address the development of behavioral/functional deficits in relation to biochemical changes temporally and assess the chronicity of changes based on the number, severity, and inter-injury interval of neurotrauma-related events. The development of better transgenic models is critical as the field moves forward. Performing studies such as these will allow for the questions raised in our accompanying review be addressed. Specifically, what is the role of the (1) inter-injury interval, (2) number of impacts, (3) impact severity, (4) age at time of impacts, (5) mechanism of impact, (6) genetics, (7) gender, and (8) effect of environment on the likelihood and/or progression of CTE development.

## Conflict of Interest Statement

The authors declare that the research was conducted in the absence of any commercial or financial relationships that could be construed as a potential conflict of interest.
